# LC-MS based identification of stylosin and tschimgine from fungal endophytes associated with *Ferula ovina*

**DOI:** 10.22038/ijbms.2020.46334.10703

**Published:** 2020-12

**Authors:** Zahra Tazik, Kamran Rahnama, James Francis White, Hassan Soltanloo, Maede Hasanpour, Mehrdad Iranshahi

**Affiliations:** 1 Department of Plant Protection, Faculty of Plant Production, Gorgan University of Agricultural Sciences and Natural Resources, Gorgan, Iran; 2 Department of Biology, Rutgers University, New Brunswick, New Jersey, U.S.A.; 3 Department of Biotechnology & Plant Breeding, Faculty of Plant Production, Gorgan University of Agricultural Sciences and Natural Resources, Gorgan, Iran; 4 Biotechnology Research Center, Pharmaceutical Technology Institute, Mashhad University of Medical Sciences, Mashhad, Iran

**Keywords:** Ferula ovina, Fungal endophytes, Ochroconis ferulica, Pithoascus persicus, Stylosin, Tschimgine

## Abstract

**Objective(s):**

*Ferula ovina* is an Iranian medicinal plant. Tschimgine and stylosin are two of its major monoterpene derivatives. In this study, we proceeded to investigate some fungal endophytes from *F. ovina* that can produce plant secondary metabolites.

**Materials and Methods:**

The isolated endophytic fungi were fermented in potato dextrose broth (PDB) medium and their extracts were screened for the presence of the plant compounds by liquid chromatography-tandem mass spectrometry (LC-MS). Endophytes identification was performed by morphological and molecular methods. Three markers (ITS, LSU, and TEF1) were used for accurate molecular identification.

**Results:**

Forty isolates from 9 different genera of endophytic fungi were identified, of which two recently reported species of *O. ferulica* and *Pithoascus persicus* were able to produce tschimgine and stylosin.

**Conclusion:**

These fungi can be used as a substitute for the production of plant’s medicinal compounds independent of wild populations of the source plant.

## Introduction

Fungi are present in all parts of plants. They may be endophytic, epiphytic, or pathogenic. The term “Endophyte” was introduced by De Bary in 1866 (1) and was initially applied to any organism found within a plant that causes asymptomatic infections entirely within plant tissues without any symptoms of disease (2). The earliest records of the presence of endophytic fungi have come from the 400-million-year-old fossils of the Early Devonian Rhynie chert deposits (3) which suggest that endophyte–plant associations may have evolved along with the evolution of higher plants (4).

Endophytes can live within their host plant for a long time. The fact that endophytic fungi are found to mimic the host plant secondary metabolite profile also led to a possibility that these plant metabolites could in fact be a product of their respective endophytes (5). Observations about the ability of plant endophytes to produce secondary plant metabolites were made about two decades ago (6, 7).

Some endophytes could be reliable sources of materials of the agricultural and/or pharmaceutical potential such as taxol (7), subglutinol A and B (8), and peptide leucinostatin A (9) (all of these could be produced by both endophytes and the hosts). Recently, several studies have led to the discovery of important plant secondary metabolites from endophytic fungi, thus, raising the prospect of using such organisms as alternative sources of these metabolites (10).

The genus *Ferula *(Apiaceae) contains more than 130 species, among which 30 species are represented in Iranian flora (11). *Ferula *species are good sources of biologically active compounds such as terpenoid, coumarins, and sesquiterpene derivatives (12, 13). *Ferula ovina* is one of the Iranian species which is an ethnomedicinal plant used in folk medicines especially in the Eastern, middle, and some Western regions of Iran. It produces valuable compounds such as stylosin and tschimgine. These two compounds are monoterpene derivatives (14). The pharmacological effects of an ester-type monoterpene, tschimgine, are cytotoxic activity against melanoma (SK-MEL-28) cell line (15), inhibition of acetylcholinesterase which is the leading strategy against Alzheimer’s disease (14), modulation the activity of estrogen receptors (ERs) (16). Stylosin also has cytotoxic and apoptotic effects (17).

This species grows in the 2000 to 3200 meters above sea level range (18).

The wild plant resource may be in short supply due to the over-collection for bioactive metabolites. Our study was therefore conducted to isolate endophytic fungi from *F. ovina* and get candidate endophytic fungal strains that produce the same bioactive compounds as the plant. For this purpose, ethyl acetate extract of these plant endophytic fungi was examined by liquid chromatography-tandem mass spectrometry (LC-MS).

## Materials and Methods

***Plant material***

**Figure 1:**
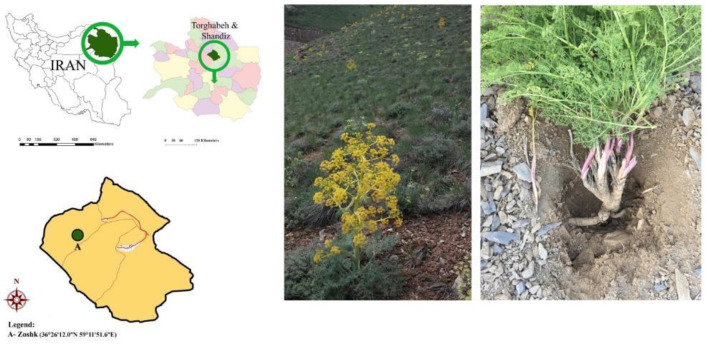
Left to Right: Map showing the locations of the sampling site (Zoshk altitudes, Khorasan Razavi, Iran), *Ferula ovina* located on a slope of the mountain during the flowering season (May 2016), Sampling from the root of the plant

Healthy root parts were collected from *F. ovina* grown in Zoshk mountains of Khorasan Razavi province, Iran (36°26′12.0″N 59°11′51.6″E) (Figure 1). Voucher specimens (No. 13274) were identified by Prof. Emami and Ms. Souzani (Department of Pharmacognosy, School of Pharmacy, Mashhad University of Medical Sciences) and were deposited at the Herbarium of the Mashhad University of Medical Sciences, School of Pharmacy, Mashhad, Iran.

***Isolation of endophytic fungi***

Isolation of endophytic fungi was carried according to the method described by Hallmann *et al.* (19). Fresh and disease-free root samples were washed with running tap water and allowed to dry. Then, they were cut into pieces of 0.5-1 cm. Root pieces were placed in ethanol 75% for 1 min and in 1-4% sodium hypochlorite solution for 3 min and then ethanol 75% for 30 sec. The samples were washed in distilled water after sterilization and placed on filter paper in sterile conditions for drying. After drying, the root parts were placed onto potato dextrose agar (PDA) and malt extract agar (MEA) media containing streptomycin (20 μg/ml) and chloramphenicol (30 μg/ml) and incubated at 25-30 ^°^C for 7–14 days. A daily survey was conducted to ensure the absence of saprophytic contamination. Hyphal tips of fungi, emerging out of the root tissues, were picked and grown on potato dextrose agar in pure culture.

Dried specimens were preserved in the Fungarium of the Iranian Research Institute of Plant Protection, Tehran, Iran (IRAN).

***Mycelial culture suspension and bioactive compound extraction***

Five agar plugs (6 mm diameter) were taken from the edges of growing colonies (3 to 5 days old) and transferred to 1000 ml Erlenmeyer flasks containing sterile potato dextrose broth (PDB) medium. Flasks were incubated at 120 rev min^-1^ on a rotary shaker at 24 ^°^C for 14 days. After finishing incubation time, mycelia were separated from the broth by filter paper. Culture filtrates were extracted three times with an equal volume of ethyl acetate. The organic phase was isolated by using a decanting funnel and evaporated using a rotary evaporator. The dried extract was kept at -20 ^°^C in a freezer. The remaining mycelia were dried and crushed with a grinder, followed by extraction with 200 ml of anhydrous ethyl acetate two times. The supernatant was isolated and its solvent was evaporated using a laboratory chemical hood. Then, the sample was lyophilized in a freeze dryer (20).

***LC–MS Screening***

The dried extract was then resolved in methanol for further LC-MS analysis and was filtered through a 0.22-μm filtration membrane. LC-MS analysis was performed in an AB SCIEX QTRAP (Shimadzu) liquid chromatography coupled with triple quadrupole mass spectrometer. Liquid chromatography separation was performed on a Supelco C18 (15 mm×2.1 mm×3 μm) column. The analysis was done at a flow-rate of 0.2 ml/min with a mixture of methanol and water (90:10), and the mass spectra were acquired in a range of 100 to 700 within the 20 min scan time. The detection was monitored at the MS-ESI (+) spectroscopy at a probe temperature of 300 ^°^C and probe voltage of 3 kV.

Several specific algorithms have been developed to detect peaks in the LC-MS chromatogram. In this study, mass feature extraction of the acquired LC-MS data and maximum detection of peaks was done using the MZmine analysis software package, version 2.3 (21).

***Morphological and molecular taxonomy***

For morphological identification, microscopic slides of the fungal isolate were prepared by staining with lactophenol cotton–blue (22) and examined under a light microscope (BX43, Olympus, Tokyo, Japan). Primary identification of the genera was done using the Ellis key for *Dematiaceous Hyphomycetes *(23).

For field emission scanning electron microscopy (FESEM) micrographs, briefly, small pieces of fungal isolates, including mycelia, conidiophores, and conidia, were placed on PELCO image tabs^™^ double-sided carbon adhesive discs (Ted Pella Inc., Redding, CA, USA), and coated with gold in a Q150R ES sputter coater (Quorum Technologies Ltd, East Sussex, United Kingdom) as previously described (24). Samples were analyzed using Field Emission Scanning Electron Microscopy (FESEM) (TESCAN BRNO-Mira3 LMU, 2014, Brno, Czech Republic) in the secondary electron imaging (SE) mode. The microscope was operated at 10 kV acceleration voltage, 1.8 kV extraction voltage, and a working distance of 4.45 mm. 

Genomic DNA of the fungal endophytes was isolated using DenaZist Asia fungal DNA isolation kit according to the manufacturer’s instructions. The DNA samples were stored at 4 ^°^C for immediate use and stored at -20 ^°^C for long-term storage. The fungal rDNA–ITS region was amplified using the fungal domain-specific ITS5 and ITS4 (25). Amplicon master mix was used to enhance amplification accuracy in a total reaction volume of 25 μl. PCR was performed in a Bio–Rad MyCycler^TM^ Thermal Cycler (Hercules, California, USA) with an initial denaturation step at 94 ^°^C for 5 min, followed by 30 cycles of 94 ^°^C for 30 sec, 58 ^°^C for 20 sec, and 72 ^°^C for 30 sec, with a final extension step of 72 ^°^C for 10 min. The primers LROR and LR5 were used to amplify the nuclear LSU gene (26). The partial translation elongation factor alpha 1 gene (TEF1) was amplified using the primers EF1-983F and Efgr (27). The amplified regions were analyzed in 1.5% agarose gel electrophoresis in 1X Tris–Boric acid–EDTA buffer (TBE) with a marker ladder of 100–bp. PCR products were sent to Macrogen Korea for sequencing. The obtained sequences were then analyzed using the BLAST algorithm and closely similar sequences obtained from the National Centre of Biological Information (NCBI) database (https://www.ncbi.nlm.nih.gov/).

## Results

***Isolation of culturable endophytic fungi from the root of Ferula ovina***

**Figure 2:**
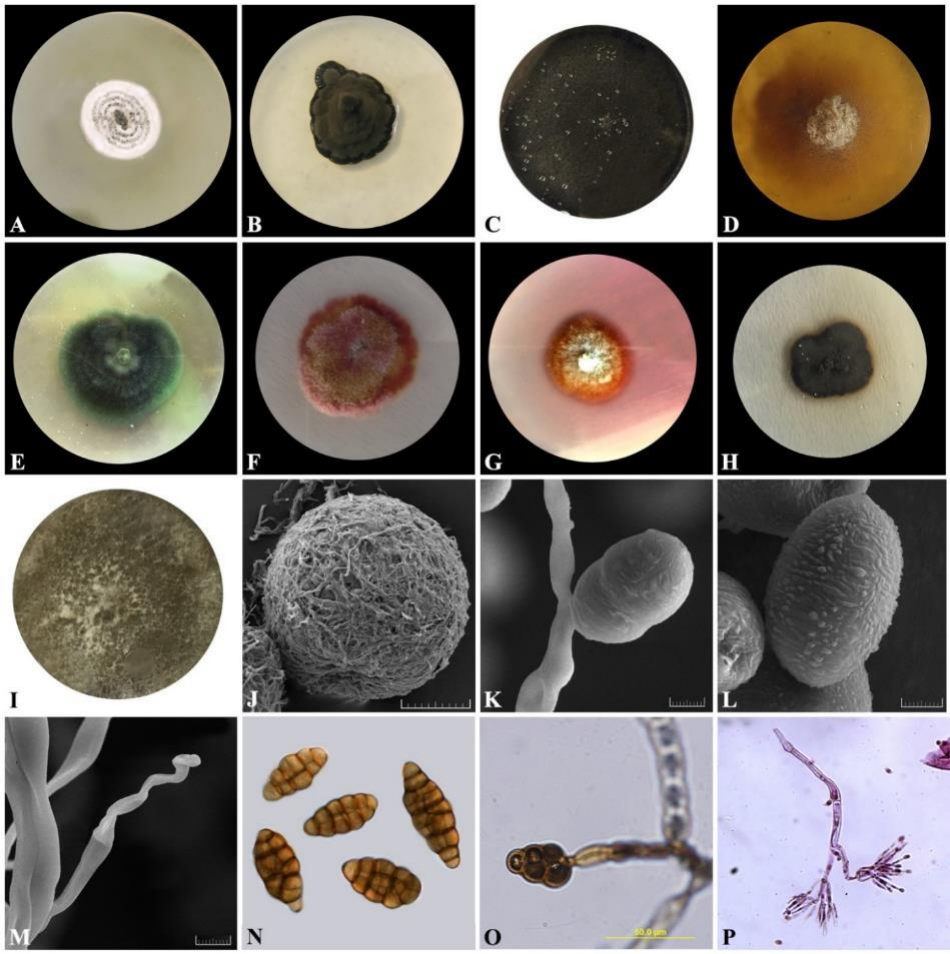
Colony on MEA of *Pithoascus persicus, Ochroconis ferulica*, and PDA of *Alternaria petroselini, Lasiobolidium* sp. Nov., unknown fungi from Lasiosphaeriaceae, *Clonostachys rosea, Laburnicola* sp. Nov., *Phaeoacremonium* sp., *Cadophora interclivum* (A-I), Scanning Electron Microscope (SEM) picture of Ascocarp of *Pithoascus persicus* (J), SEM pictures of Conidia and sympodial Conidiophore of *Ochroconis ferulica *(K-M), Conidia and Conidiophore of *A. petroselini *(N, O), Conidiophore of C. rosea (P). Scale bars: 50 µm (J, O), 2 µm (K, L, and M)

Since molecular identification of fungal isolates was not possible using only ITS rDNA regions, the LSU region and part of the protein-coding gene EF1–α were also sequenced. Based on the BLASTn results of the three gene regions, 40 isolates from 9 different genera of endophytic fungi were identified all of which belonged to *Ascomycota* phylum, *Pezizomycotina* subphylum but in 4 different classes. The molecular taxonomy was also supported by morphology investigation (Figure 2). Name, NCBI accession numbers, and voucher numbers of isolated fungal endophytes are listed in Table 1.

***LC-MS based screening for Plant medicinal compounds Production***

**Figure 3:**
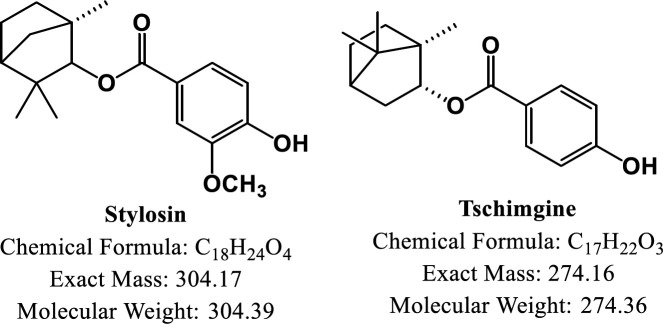
Molecular structures and chemical information of stylosin and tschimgine

In the current study, sensitivity of the LC-MS approach has been effectively investigated for the identification of secondary metabolites found in the endophytic fungal species. Stylosin (C_18_H_24_O_4)_ and tschimgine (C_17_H_22_O_3_) are two known major compounds in the roots of *F. ovina* (Figure 3) that have been received considerable attention towards their potential biological activities. 

**Figure 4:**
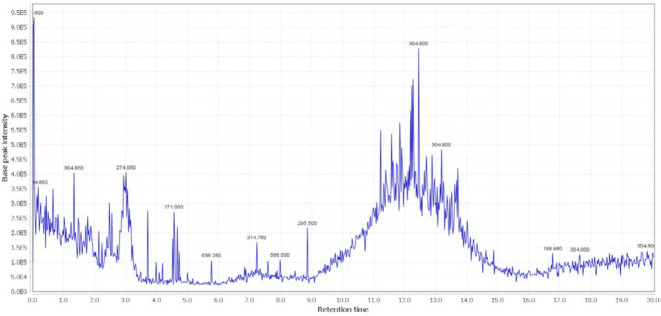
The total ion chromatogram (TIC) of the endophytic fungal extract isolate *Ochroconis ferulica* obtained by LC-MS analysis

**Figure 5:**
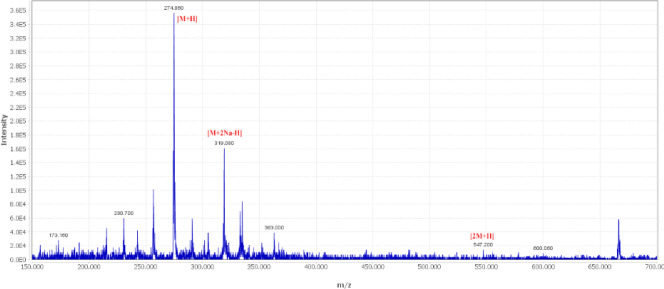
Mass spectra of tschimgine and corresponding adducts detected in the endophytic fungal extract isolate *Ochroconis ferulica*. The detection was monitored at MS-ESI (+) spectroscopy at a probe temperature of 300 ^°^C and probe voltage of 3 kV

**Figure 6:**
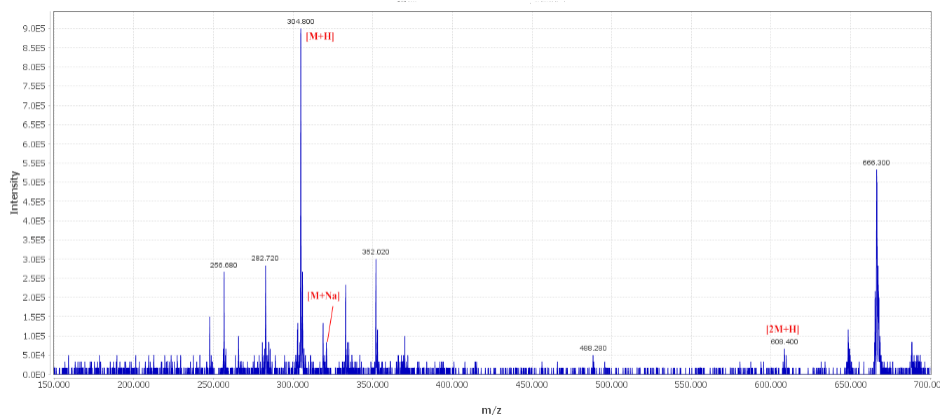
Mass spectra of stylosin and corresponding adducts detected in the endophytic fungal extract isolate *Ochroconis ferulica*. The detection was monitored at the MS-ESI (+) spectroscopy at a probe temperature of 300 ^°^C and probe voltage of 3 kV

**Figure 7:**
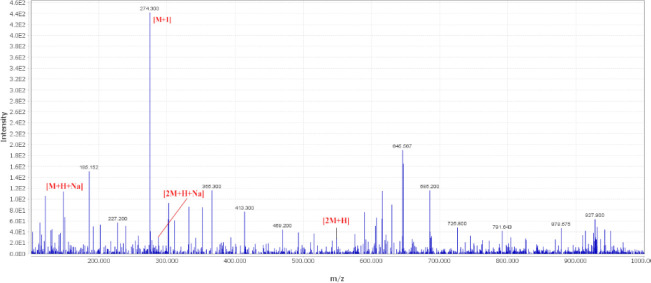
Mass spectra of tschimgine and corresponding adducts detected in the endophytic fungal extract isolate *Pithoascus persicus*. The detection was monitored at MS-ESI (+) spectroscopy at a probe temperature of 300 ^°^C and probe voltage of 3 kV

**Figure 8:**
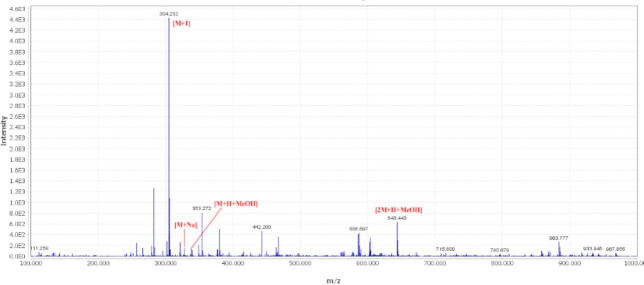
Mass spectra of stylosin and corresponding adducts detected in the endophytic fungal extract isolate *Pithoascus persicus*. The detection was monitored at MS-ESI (+) spectroscopy at a probe temperature of 300 ^°^C and probe voltage of 3 kV

The total ion chromatogram (TIC) of the endophytic fungal extract is shown in Figures 4-8, which indicates the ability of these two endophytic fungal species to produce some coexisting plant secondary metabolites.

Both stylosin and tschimgine were detected from both endophytic fungal extracts. The ethyl acetate extracts of *Ochroconis ferulica *(28) and *Pithoascus persicus *(29) were analyzed using the LC-MS method and positive mode ionization of the mass spectrometer. The total ion chromatogram of *O. ferulica* extract is shown in Figure 4. Tschimgine (t_R_=3.0 min) displayed molecular ion [M]^+ ^and protonated molecule [M+H]^+^ at *m/z* 274.86 and 275.76, respectively (Figure 5). The adduct ion [2M+H]^+^ at *m/z* 547.20 was successfully detected in the mass spectrum. Stylosin peak at the retention time of 12.5 min was detected in the total ion chromatogram of endophytic fungal extract isolate *O. ferulica* (Figure 4). This compound showed molecular ion [M]^+^ and protonated molecule [M+H]^+^ at *m/z* 304.80 and 305.76, respectively (Figure 6). The sodium adduct [M+Na]^+ ^and protonated dimmer adduct ion [2M+H]^+ ^of stylosin were also observed at *m/z *327.82 and 609.66, respectively (Figure 6). 

The ethyl acetate extract of *P. persicus *was also analyzed by the LC-MS method. The mass spectra of two peaks at the retention times of 3.5 and 9.3 min are shown in Figure 7 and Figure 8 which are respectively related to the production of tschimgine and stylosin by this endophytic fungus. The molecular ion [M]^+ ^and protonated molecule [M+H]^+^ signals of tschimgine (t_R_=3.5 min) were detected at *m/z* 274.30 and 275.33, respectively (Figure 7). Some of its related adduct including sodium adduct ([M+H+Na] and [2M+H+Na]^+^ and protonated dimmer adduct ion [2M+H]^+ ^were respectively observed at *m/z* 148.3, 286.9, and 546.3. Stylosin (t_R_=9.3 min) displayed molecular ion [M]^+^ and protonated molecule [M+H]^+^ signals at *m/z* 274.86 and 275.76, respectively (Figure 8). The sodium adduct of stylosin was observed at *m/z* 327.21. 

***Morphological characteristics of endophytic fungi producing stylosin and tschimgine***

*Ochroconis ferulica*

Z. Tazik & K. Rahnama (2020)

Classification: Ascomycota, Pezizomycotina, Dothideomycetes, Venturiales, Sympoventuriaceae, *Ochroconis*

After 2 weeks in darkness, on malt extract agar (MEA) medium at 24 ^°^C, colonies were velvety to floccose with some shallow radial fissures, dark olive-brown to blackish brown with a submerged irregular margin and grew slowly to attain a diameter of 8 mm [Figure 3 (B)]. The colonies were black on the reverse side. The hyphae were dark brown, smooth, with thick walls melanized. There was no detection of hyphal coils. Conidiophores were dark brown, bearing one or more conidia, with a sympodial proliferating conidiogenous locus, arising individually from vegetative hyphae with right angles, erect, cylindrical with 1-2 septa, 25-30×2-2.5 μm. Conidia were pale brown, mostly 2-celled, rarely single, 3-celled and 4-celled, sometimes slightly apiculate ellipsoidal, 8-10.5 × 6-7.5 μm, not or slightly restricted at the septa, rough-walled, with a thick median septum [Figure 3 (K, L, M)].

*Pithoascus persicus*

Z. Tazik & K. Rahnama (2020).

Classification: Ascomycota, Pezizomycotina, Sordariomycetes, Hypocreomycetidae, Microascales, Microascaceae, *Pithoascus*

Colony on MEA medium was creamy white somewhat velvety and very slow-growth attained a diameter of 23 mm at 25 ^°^C after 2 weeks [Figure 3 (A)]. Dark ascocarps were abundantly seen and often form dense crusts, dark brown to black, glabrous, non-ostiolate with a diameter of 200–260 μm [Figure 3 (J)]. Asci were thin-walled labile. Single-cell ascospores were abundantly observed with an almost lunate, yellowish, flat, boat-shaped, 6.5-8×3.5-3.8 μm.

**Table 1:** *Ferula ovina* endophytes with their voucher numbers and GenBank accession numbers

**Isolate code**	**Fungi name**	**Family**	**Voucher number**	**Gene bank accession numbers**		
				**ITS**	**LSU**	**TEF1**
AT01	*Pithoascus* *persicus*	Microascaceae	IRAN3309C	MF186873	MH400206	MK430530
AT02	*Ochroconis* *ferulica*	Sympoventuriaceae	IRAN3232C	MF186874	MH400207	MK512743
FO1	*Alternaria petroselini*	Pleosporaceae	IRAN3310C	MF186875	MH400221	MK512744
FO51	*Lasiobolidium* sp. Nov.	-	-	MK312604	MH400222	-
FO61	Unknown fungi	Lasiosphaeriaceae	-	-	MH400223	MK512745
FO7	*Clonostachys rosea*	Bionectriaceae	IRAN3313C	MH458900	-	-
FO8	*Laburnicola *sp.Nov.	Didymosphaeriaceae	IRAN3311C	MF186878	MH400224	MK512746
FO9	*Phaeoacremonium* sp.	Togniniaceae	IRAN3312C	MF186879	MH400225	MK512747
FO11	*Cadophora interclivum*	-	IRAN3316C	MF186880	MH400226	MK512748

ITS: Internal transcribed spacer; LSU: large ribosomal subunit; TEF1: Translation Elongation factor 1-α

## Discussion

Of all the plants in the world, only a few grass species were thoroughly studied for their endophytes. Thus, it is of significance to find interesting strains among a variety of plants (30). *Ferula* species have been used in folk medicine. They are a good source of biologically active compounds such as sesquiterpene derivatives which are stored in the roots of the plants (31-35, 12).

Researchers studied the diversity of endophytic fungi associated with *Ferula sinkiangensis* in 2014. 140 endophytic fungi were isolated. Among them, *Aureobasidium* (25.7%), *Alternaria* (16.4%), and *Phyllosticta* (15.7%) were the dominant genera (36). 

A recent study on endophytic fungi residing in *Ferula sumbul *has led to the discovery of some new biologically active metabolites with potential anti-cancer and anti-microbial activities (37).

The endophytic fungi isolated from *F. ovina* have not been previously reported from other *Ferula* species, four of which are new fungal species (Table 1).

The identification of stylosin and tschimgine metabolites in the endophytic fungal extract isolate *O. ferulica* indicate the ability of this new fungal species to produce two major secondary metabolites of *F. ovina *roots.

## Conclusion

The genus *Ferula* comprises about 170 species (38), of which only endophytic fungi of three species have as yet been studied. Two recently reported endophytic fungi, namely, *O. ferulica* and *P.* *persicus*, have been isolated from the roots of *F. ovina*. The results of the LC–MS spectra analysis confirm that these two new species of *Ochroconis* and *Pithoascus* are able to produce two main plant chemical compounds. The results of the present and past research indicate the high capacity of species of this genus to identify and introduce new endophytic fungi and new metabolites.
